# Cerebrospinal fluid N-224 tau helps discriminate Alzheimer’s disease from subjective cognitive decline and other dementias

**DOI:** 10.1186/s13195-020-00756-6

**Published:** 2021-02-08

**Authors:** Claudia Cicognola, Oskar Hansson, Philip Scheltens, Hlin Kvartsberg, Henrik Zetterberg, Charlotte E. Teunissen, Kaj Blennow

**Affiliations:** 1 Clinical Memory Research Unit, Department of Clinical Sciences, Lund University, Lund, Sweden; 2 Memory Clinic, Skåne University Hospital, Malmö, Sweden; 3 Department of Neurology, Alzheimer Center Amsterdam, Amsterdam Neuroscience, Amsterdam UMC, Vrije Universiteit, Amsterdam, the Netherlands; 4 Clinical Neurochemistry Laboratory, Sahlgrenska University Hospital, Mölndal, Sweden; 5 Department of Psychiatry and Neurochemistry, Institute of Neuroscience and Physiology, The Sahlgrenska Academy, University of Gothenburg, Mölndal, Sweden; 6 Department of Neurodegenerative Diseases, UCL Institute of Neurology, London, UK; 7 UK Dementia Research Institute at UCL, London, UK; 8 Neurochemistry Laboratory, Department of Clinical Chemistry, Amsterdam Neuroscience, Amsterdam UMC, Vrije Universiteit, Amsterdam, the Netherlands

**Keywords:** Tau, Biomarkers, Alzheimer’s disease, Subjective cognitive decline, Parkinsonisms

## Abstract

**Background:**

Elevated cerebrospinal fluid (CSF) concentrations of total tau (T-tau) and phosphorylated tau at Thr181 (P-tau181) protein are typical of Alzheimer’s disease (AD). However, the T-tau assay measures only the mid-region of the protein, while tau in CSF is instead composed of a series of fragments. One fragment species in particular, N-224, shows increased levels in AD compared to controls. In this multicentre study, we performed a clinical validation of the N-224 assay in cohorts including patients with subjective cognitive decline (SCD), mild cognitive impairment (MCI), AD, non-AD dementias and controls.

**Methods:**

Cohorts consisted of 30 SCD and 30 probable AD from the Amsterdam Dementia Cohort (cohort 1) and 539 controls, 195 SCD, 232 MCI, 137 AD and 253 non-AD from the Swedish BioFINDER study (cohort 2). All samples had AD core biomarkers (Aβ42, T-tau, P-tau181) measurements. N-224 was measured with an in-house ultrasensitive Simoa assay.

**Results:**

N-224 levels were significantly higher in AD compared to SCD (cohort 1: *p* = 0.003) and in AD compared to all other diagnostic groups in cohort 2 (control, SCD, MCI and non-AD, *p* < 0.0001). Within the non-AD group, N-224 showed significantly lower concentrations compared to AD in Parkinson’s disease (PD, *p* < 0.0001), Parkinson’s disease dementia (PDD, *p* = 0.004), progressive supranuclear palsy (PSP, < 0.0001), multiple system atrophy (MSA, *p* = 0.002) and parkinsonisms not otherwise specified (NOS, *p* = 0.007). In cohort 1, higher concentrations of N-224 were associated to lower Mini-Mental State Examination (MMSE) scores (*R*^2^ = 0.318, *β* = 0.564, *p* ≤ 0.0001) and could accurately identify a pathological (< 24) MMSE score (*p* < 0.0001, AUC = 0.824).

**Conclusions:**

N-224 tau can distinguish AD subjects from SCD and can discriminate subgroups of non-AD dementias from AD. Therefore, N-224 may be a useful addition to the tau biomarker toolbox for the study of tau species in CSF and for better understanding disease pathogenesis.

## Background

Tau pathology, in the form of neurofibrillary tangles (NFT) of hyperphosphorylated tau, is one of the hallmarks of Alzheimer’s disease (AD), together with amyloid β (Aβ) plaques. Tau protein can be measured in cerebrospinal fluid (CSF) as total tau (T-tau), which is increased in AD and other neurological diseases, and as phosphorylated tau (P-tau181), which is specifically increased in AD; Aβ42, on the other hand, shows decreased CSF levels [1]. These changes occur up to decades before the onset of clinical symptoms, with changes in Aβ42 appearing first, followed by P-tau181 and T-tau [2]. These proteins are typically measured in CSF by means of immunoassays (enzyme-linked immunosorbent assay, ELISA). Traditional tau immunoassays, in particular, target the mid-region of the protein (T-tau) or a single phosphorylation site (Thr181). However, several studies have shown that tau in CSF is very heterogeneous and is present not as a full-length protein but as a series of fragments belonging mostly to the N-terminal and mid region of the protein, rather than the C-terminal [3–8]. In a recent study, we have observed that a relatively abundant pool of tau species in CSF ends at amino acid (aa) 224, which can be measured with an ultrasensitive immunoassay (single molecule array, Simoa) [3]. These tau fragments, spanning the N-terminal to aa 224 (N-224), were significantly more abundant in CSF from AD subjects compared to healthy elderly controls or subjects with other neurological diseases. The CSF concentrations of the fragments were not correlated to those of T-tau in primary tauopathies such as progressive supranuclear palsy (PSP) and corticobasal syndrome (CBS), suggesting that quantifying N-224 in these diseases could help in the differential diagnosis with AD, as classic tau biomarkers are often normal in non-AD dementias [9–11]. Taken together, these results suggested that the increase in N-224 was specific for AD, and we wanted to validate these findings in independent cohorts including cognitively healthy subjects and patients with cognitive impairment unrelated to AD.

Patients referred to memory clinics are often initially diagnosed with *subjective cognitive decline* (SCD). SCD is defined as self-perception of cognitive decline that is not confirmed by objective cognitive tests [12]. It is still unclear which type of relationship stands between SCD and early-stage AD, and what percentage of SCD patients will progress to MCI or dementia. Even though there is no objective proof of cognitive impairment, at least part of these individuals are at increased risk of cognitive decline; at the same time, they may also not eventually show further progression in the cognitive decline or evolve into overt AD [13].

Differential diagnosis between SCD and AD or other dementias at early stages is problematic: studies on levels of CSF Aβ42, T-tau and P-tau181 in SCD have shown mixed results and no studies are available on CSF tau fragments in SCD patients [14–17].

The aim of the study was to perform a clinical validation of the N-224 tau assay in a multicentre study with two independent cohorts, one including SCD and probable AD (cohort 1) and one including cognitively healthy controls, SCD, mild cognitive impairment (MCI), and patients with AD and non-AD dementias (cohort 2).

## Methods

### Study population

Cohort 1 included 60 subjects from the Amsterdam Dementia Cohort [18, 19] with a baseline clinical diagnosis of SCD (*n* = 30) or probable AD (*n* = 30) (Table 1). Subjects visited the Amsterdam Alzheimer center between July 2009 and March 2017 for standardized dementia screening consisting of neurological, physical and neuropsychological evaluation and brain magnetic resonance imaging (MRI) [18, 19]. Global cognition was assessed by the Mini-Mental State Examination (MMSE) and subjects were genotyped for *APOE*. Diagnoses were made in a multidisciplinary consensus meeting according to the then applicable guidelines [20–23]. The label of SCD was assigned when no abnormalities were observed on clinical or cognitive tests and when the criteria for MCI, dementia, or other medical conditions and psychiatric disorders that could potentially cause cognitive deficits were not met. Clinical diagnosis of probable AD was established according to National Institute on Aging and Alzheimer’s Association (NIA-AA) criteria [23]. Cohort 2 (Table 1) consisted of patients enrolled in the prospective Swedish BioFINDER study (Lund, Sweden). Only baseline cross-sectional data are included in this study. The inclusion criteria for control individuals (*n* = 539) were as follows: (1) absence of cognitive symptoms as assessed by a physician with special interest in cognitive disorders, (2) MMSE 28–30 points at screening visit, (3) did not fulfil the criteria for MCI or any dementia disorder and (4) fluency in Swedish. The exclusion criteria were (1) significant unstable systemic illness, such as terminal cancer, or organ failure that made it difficult to participate in the study; (2) current significant alcohol or substance misuse; and (3) significant neurological or psychiatric illness. The inclusion criteria for subjects with SCD (*n* = 195) and MCI (*n* = 232) were as follows: (1) referral to the memory clinic due to cognitive symptoms experienced by the patient and/or an informant; (2) criteria of any dementia disorder not fulfilled; (3) MMSE score 24–30; (4) age 60–80 years; and (5) fluency in Swedish. Exclusion criteria were (1) significant unstable systemic illness or organ failure, (2) current significant alcohol or substance misuse and (3) cognitive impairment that could be explained by other specific non-neurodegenerative disorders such as brain tumour or subdural hematoma. The classification into SCD or MCI was based on a neuropsychological battery and the clinical assessment of a senior neuropsychologist as previously described [24]. AD dementia patients (*n* = 137) fulfilled the Diagnostic and Statistical Manual of Mental Disorders-5 (DSM-5) criteria for major neurocognitive disorder (dementia) due to AD. The non-AD group (*n* = 253) included patients with vascular dementia (VaD, *n* = 12), Parkinson’s disease dementia (PDD, *n* = 25), dementia with Lewy bodies (DLB, *n* = 21) and frontotemporal dementia (FTD, *n* = 5), all fulfilling the respective DSM-5 criteria. The group also included Parkinson’s disease (PD) subjects (*n* = 128, fulfilling the criteria by Gelb et al. [25]), PSP(*n* = 17, fulfilling the criteria by Litvan et al. [26] and Höglinger et al. [27]), multiple system atrophy (MSA, *n* = 27, fulfilling the criteria by Gilman et al. [28]) and CBS (*n* = 3, fulfilling the criteria by Armstrong et al. [29]). Other patients had dementia or parkinsonisms not otherwise specified (NOS, *n* = 4 and 11, respectively). CSF biomarker measurements and *APOE* genotyping were performed at baseline.

**Table 1:** Demographic and clinical data from (a) cohort 1 and (b) cohort 2. Values are expressed as mean (range)

**(a) Cohort 1**		**SCD**		**Probable AD**	
*N*		30		30	
Age		57 (38–78)		69 (48–80)	
Gender (m/f)		18/12		11/19	
At least one *APOE* ε4 allele		29.6%		57.1%	
MMSE		28.48 (25–30)		19 (9–29)	
N-224 (pg/ml)		65 (12–184)		194 (33–704)	
Aβ42 (pg/ml)		1052 (656–1550)		597 (304–810)	
T-tau (pg/ml)		260 (68–527)		711 (239–1776)	
P-tau181 (pg/ml)		47 (12–92)		93 (42–252)	
**(b) Cohort 2**	**Control**	**SCD**	**MCI**	**AD**	**Non-AD**
*N*	539	195	232	137	253
Age	72 (41–88)	71 (60–81)	72 (60–81)	74 (52–88)	68 (39–87)
Gender (m/f)	185/354	92/103	133/99	49/88	157/96
At least one *APOE* ε4 allele	30.8%	39.9%	47.8%	68.1%	31.8%
N-224 (pg/ml)	63 (4–1251)	53 (6–230)	71 (6–488)	119 (7–666)	50 (6–483)
Aβ42/40 (pg/ml)	0.12 (0.03–0.23)	0.11 (0.04–0.20)	0.09 (0.03–0.21)	0.06 (0.02–0.09)	0.11 (0.02–0.19)
T-tau (pg/ml)	324 (100–1297)	349 (89–1658)	420 (46–1427)	652 (265–1448)	325 (112–926)
P-tau181 (pg/ml)	44 (15–240)	54 (7–256)	72 (14–257)	122 (18–251)	42 (13–179)

### CSF collection and core biomarker analysis

CSF was collected according to standardized procedures [1]. In cohort 1, levels of Aβ42, T-tau and P-tau181 were quantified using Innotest ELISAs (Fuijirebio) (Table 1). CSF Aβ42 levels were corrected to counteract a drift that occurred over the years explained by the analytical procedure [30]. Cut-offs for a biomarker-positive CSF profile were Aβ42 < 813 pg/ml, T-tau > 375 pg/ml and P-tau181 > 52 pg/ml. In cohort 2, CSF Aβ42, Aβ40, T-tau and P-tau181 were measured using ELISA (Euroimmun) according to the manufacturer’s recommendations (Table 1). A CSF Aβ42/40 ratio < 0.09 (calculated by Youden index within the cohort) was defined as pathological.

### Simoa assay targeting tau N-224

Identification of the N-224 tau fragment and development of the targeted immunoassay were previously described in Cicognola et al. [3]. Briefly, tau fragments ending at aa 224 were observed in CSF after enrichment by immunoprecipitation (IP) using tau antibodies targeting the N-terminal (Tau12, Nordic Biosite) and mid-region of the protein (HT7, BT2, both Thermo Scientific) followed by detection with mass spectrometry (MS). Monoclonal antibodies targeting the aa 224 site were developed in-house by immunization of 8-week-old Balb/c mice with a KLH-conjugated peptide (sequence: KLH-CGGGRTPSLPTPPTREPK, corresponding to aa position 207–224), amplified in myeloma cells and screened against full-length recombinant tau and recombinant protein fragments. Clones that reacted with the recombinant protein fragments but not with full-length tau and negative control protein were further grown. The anti-224 antibody was further tested with IP-MS in CSF where it exclusively pulled down tau fragments ending at aa 224 [3]. For the N-224 Simoa assay, magnetic beads (Quanterix, Billerica, MA) were conjugated with the capture antibody anti-Tau 224 according to bead supplier’s conjugation protocol. Prior to each run, Tau 224 recombinant protein calibrator was serially diluted and the biotin-labelled antibody Tau 12 (Nordic Biosite, Täby, Sweden) was used for detection. The assay showed a 10% cross-reactivity with spiked-in recombinant tau fragment ending at aa 368.

### Statistical analysis

Data were analysed using SPSS for Windows version 26 (IBM) and R v4.0.3. Graphs were constructed using SPSS or GraphPad Prism 8.3.1. Biomarkers concentrations were LOG (Log10) transformed prior to linear regression and univariate general linear model statistical analyses. All models were adjusted for age and gender. Logistic regression was used with AD diagnosis and MMSE score < 24 as outcome variables. Probabilities from logistic regression models for combinations of biomarkers were saved as variables. Akaike information criterion (AIC) was calculated for logistic regression models, with a smaller number indicating a better model. Receiver operating characteristic (ROC) curves were constructed to test the accuracy of individual biomarkers and their combinations in identifying probable AD subjects and subjects with MMSE score < 24. AUCs were compared using DeLong test. Correlations between different biomarkers and age were assessed using Spearman’s correlation.

## Results

### Demographics, clinical information and correlations

Demographics and clinical information for cohorts 1 and 2 are shown in Table 1a and b respectively). Four SCD subjects had at least pathological Aβ42 in cohort 1 while, in cohort 2, 128 controls (23.7%), 66 SCD (33.8%), 129 MCI (55.6%) and 62 non-AD (24.5%) had pathological CSF Aβ42/40. N-224 correlated significantly with age in the SCD cohorts (cohort 1: *r* = 0.647, *p* < 0.0001; cohort 2: *r* = 0.311, *p* < 0.0001) and other groups except AD (cohort 2, controls: *r* = 0.182, *p* < 0.0001; MCI: *r* = 0.164, *p* = 0.012; non-AD: *r* = 0.173, *p* = 0.006). N-224 strongly correlated with T-tau and P-tau181 in all cohorts and groups (Supplementary Table 1a, b).

### Group differences for N-224 and core AD biomarkers

**Fig. 1:**
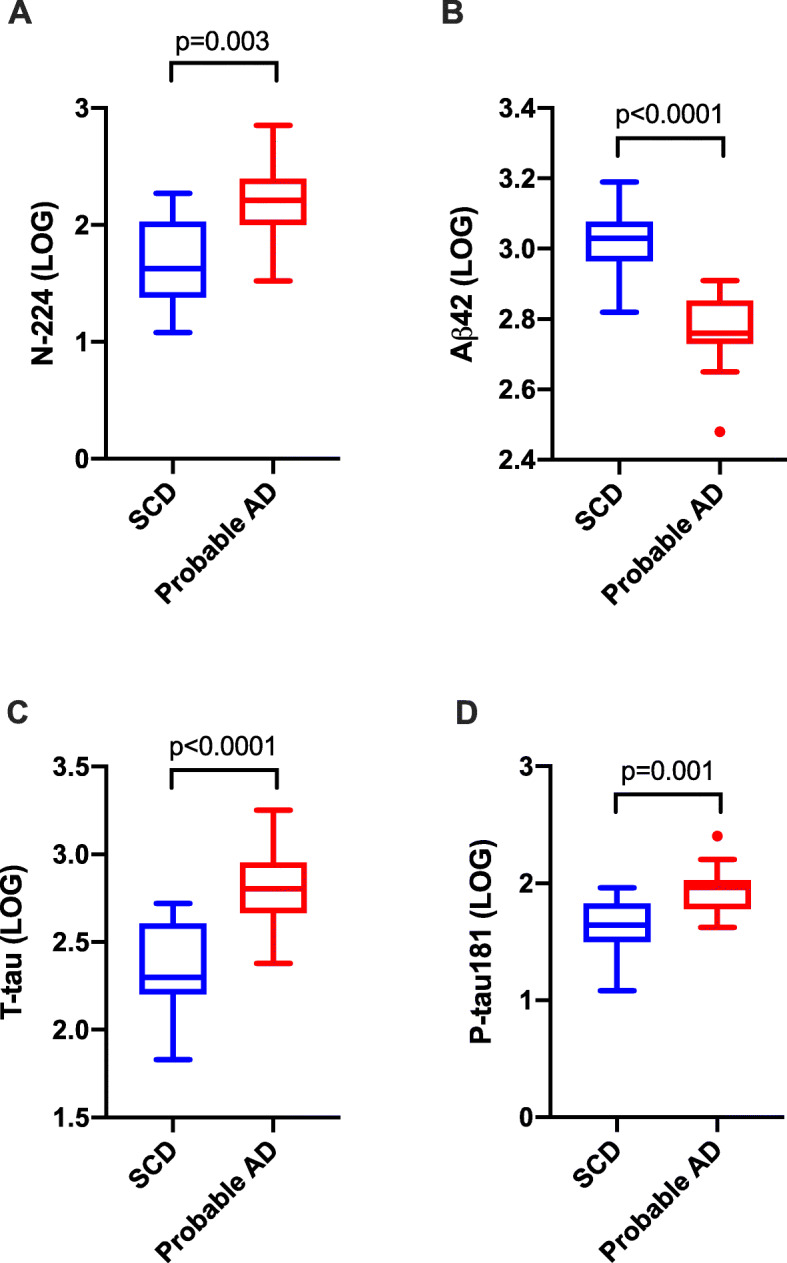
Concentrations (LOG) of N-224 (**a**), Aβ42 (**b**), T-tau (**c**) and P-tau181 (**d**) in cohort 1. *p* values of differences between subjective cognitive decline (SCD) and probable Alzheimer’s disease (AD) groups are shown above groups. Lines across represent median, boxes represent interquartile range (IQR) and bars represent min and max value (within ± 1.5 IQR)

**Fig. 2:**
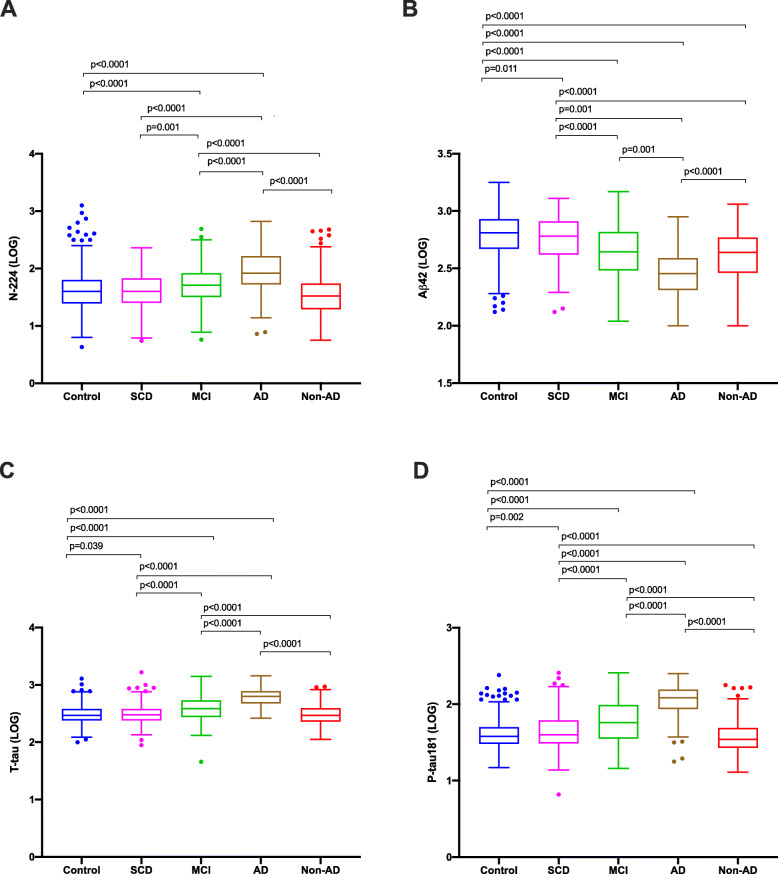
Concentrations (LOG) of N-224 (**a**), Aβ42 (**b**) T-tau (**c**) and P-tau181 (**d**) in cohort 2. *p* values of differences between controls, subjective cognitive decline (SCD), mild cognitive impairment (MCI), Alzheimer’s disease (AD) and non-AD groups are shown above groups. Lines across represent median, boxes represents interquartile range (IQR) and bars represent min and max value (within ± 1.5 IQR)

In cohort 1, there was a significant difference for N-224 between SCD and probable AD (*p* = 0.003) (Fig. 1a). AD core biomarkers also showed a significant difference between SCD and probable AD (Aβ42: *p* < 0.0001; T-tau: *p* < 0.0001; P-tau181: *p* = 0.001) (Fig. 1b–d). In cohort 2, N-224 was significantly higher in AD compared to every other diagnostic group (*p* < 0.0001 for each group comparison) (Fig. 2a). No differences were observed between the control and SCD groups. Core AD biomarkers were significantly higher (T-tau, P-tau181) or lower (Aβ42) in AD compared to every other group (*p* < 0.0001 for all three) and in SCD compared to controls (Aβ42, *p* = 0.011; T-tau, *p* = 0.039; P-tau181, *p* = 0.002) (Fig. 2b–d).

**Fig. 3:**
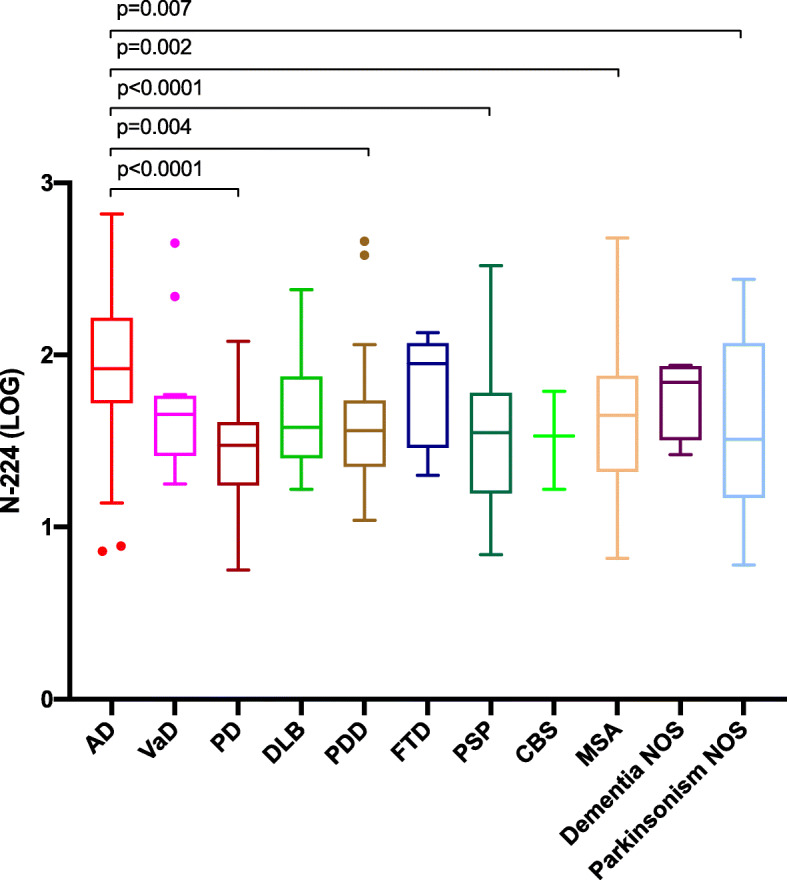
Concentrations (LOG) of N-224 in Alzheimer’s disease (AD) compared to non-AD diseases. Lines across represent median, boxes represent interquartile range (IQR) and bars represent min and max value (within ± 1.5 IQR). VaD, vascular dementia; PD, Parkinson’s disease; DLB, dementia with Lewy bodies; PDD, Parkinson’s disease dementia; FTD, frontotemporal dementia; PSP, progressive supranuclear palsy; CBS, corticobasal syndrome; MSA, multiple system atrophy; NOS, not otherwise specified

When looking at individual non-AD conditions, N-224 showed significantly higher concentrations in AD compared to PD (*p* < 0.0001), PDD (*p* = 0.004), PSP (< 0.0001), MSA (*p* = 0.002) and parkinsonism NOS (*p* = 0.007) (Fig. 3). T-tau and P-tau181 were significantly higher in AD compared to each non-AD group.

### Accuracy of N-224 and core biomarkers for identification of AD status

**Fig. 4:**
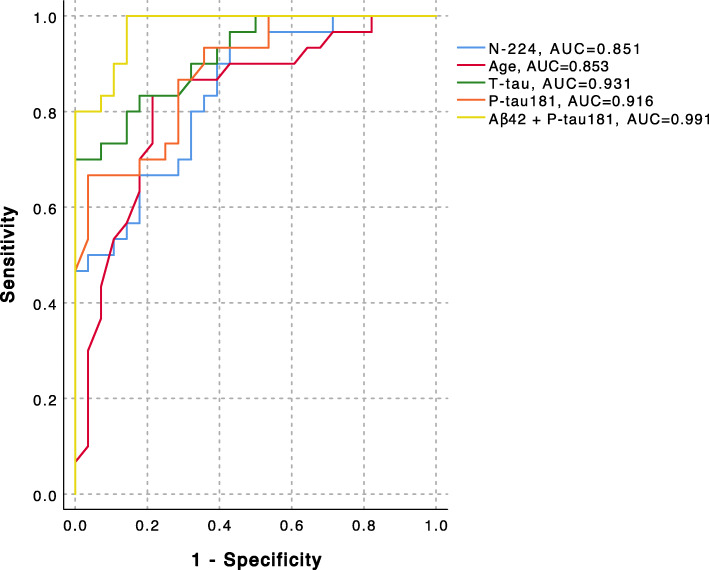
Receiver operating characteristic (ROC) curve analyses for distinguishing the Alzheimer’s disease (AD) group from subjective cognitive decline (SCD) group in cohort 1. AUC, area under the curve

**Fig. 5:**
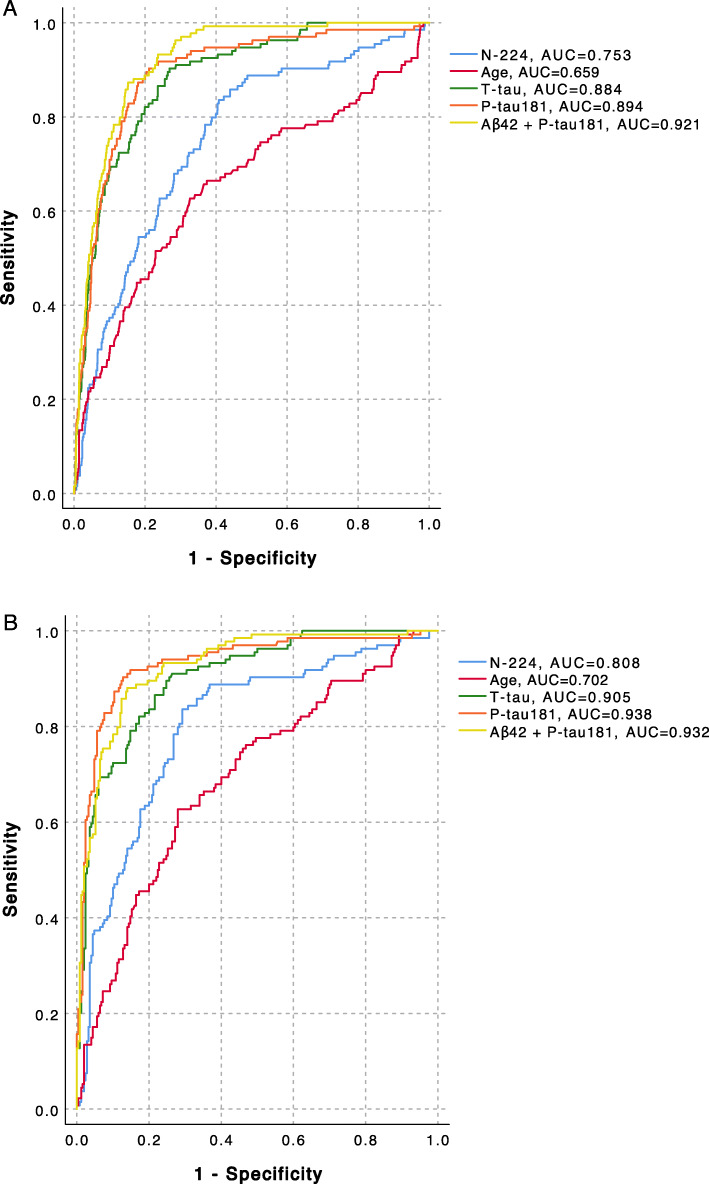
Receiver operating characteristic (ROC) curve analyses for distinguishing the Alzheimer’s disease (AD) group from controls, subjective cognitive decline (SCD), mild cognitive impairment (MCI) and non-AD group combined (**a**) or from non-AD only (**b**). AUC, area under the curve

In cohort 1, ROC curve analysis showed Aβ42 combined with P-tau181 as the best indicator of AD status (AUC = 0.991, AIC = 17.8) (Fig. 4, Table 2). In this cohort, N-224 had the lowest AUC (0.851) compared to the other biomarkers (Table 2). In cohort 2, N-224 was a better indicator of AD status than age both when considering controls, SCD, MCI and non-AD combined (*p* = 0.007) or non-AD only (*p* = 0.002) (Tables 3 and 4). The combination of Aβ42 and P-tau181 had the highest accuracy in indicating AD diagnosis (AUC = 0.921, AIC = 593.3). When comparing AD to non-AD only, P-tau181 was the most accurate biomarker for identifying AD, by itself (AUC = 0.938, AIC = 228.6) or combined with N-224 (AUC = 0.936, AIC = 266.5) or Aβ42 (AUC = 0.932, AIC = 257.9) (Fig. 5b, Table 4). The combination of N-224 with information on *APOE* status (presence of at least one ε4 allele) did not improve the accuracy of N-224 in any of the cohorts (Tables 2, 3 and 4). Adding T-tau and P-tau181 to the model significantly improved the accuracy compared to N-224 alone (*p* < 0.0001), but the combination of N-224 with T-tau and P-tau did not show superior accuracy to T-tau and P-tau181 by themselves. Logistic regression models for N-224/T-tau and N-224/P-tau181 ratios for indication of AD status were not significant. ROC curve analysis for the ratios showed lower AUCs compared to the other biomarkers in both cohort 1 (N-224/T-tau, AUC = 0.568; N-224/P-tau181, AUC = 0.715) and cohort 2 for AD compared to all other groups (N-224/T-tau, AUC = 0.554; N-224/P-tau181, AUC = 0.407) and AD compared to non-AD (N-224/T-tau, AUC = 0.623; N-224/P-tau181, AUC = 0.428). *p* values from binary logistic regression with AD status as outcome are shown in Tables 2, 3 and 4. 95% confidence intervals (95% CI) of AUCs and differences between ROC curves are also shown in Tables 2, 3 and 4.

**Table 2:** Cohort 1. *P*-values and Akaike information criterion (AIC) from binary logistic regression. Area under the curve (AUC), 95% confidence intervals (95% CI) and differences between AUCs measured with DeLong test from receiver operating characteristic (ROC) analysis. *APOE* stands for presence of at least one *APOE* ε4 allele

Cohort 1	p	AIC	AUC	95%CI	*p* value for difference with model containing N-224 alone
N-224	< 0.0001	54.4	0.851	0.751–0.952	–
Age	< 0.0001	53.6	0.853	0.743–0.963	ns
T-tau	< 0.0001	39.9	0.931	0.870–0.993	0.005
P-tau181	< 0.0001	43.9	0.916	0.846–0.987	ns
N-224+T-tau	< 0.0001	38.8	0.944	0.889–0.999	ns
N-224+P-tau181	< 0.0001	47.2	0.919	0.850–0.987	ns
N-224+*APOE*	< 0.0001	54.1	0.873	0.782–0.964	ns
Aβ42+P-tau181	< 0.0001	17.8	0.991	0.973–1.010	0.004

**Table 3:** Cohort 2. Alzheimer’s disease (AD) group compared to controls, subjective cognitive decline (SCD), mild cognitive impairment (MCI) and non-AD groups combined. *p* values and Akaike information criterion (AIC) from binary logistic regression models. Area under the curve (AUC), 95% confidence intervals (95% CI) and differences between AUCs measured with DeLong test from receiver operating characteristic (ROC) analysis. *APOE* stands for presence of at least one *APOE* ε4 allele

	Cohort 2	*p*	AIC	AUC	95%CI	*p* value for difference with model containing N-224 alone
**AD vs. controls, SCD, MCI and non-AD**	N-224	< 0.0001	823	0.753	0.709–0.797	–
	Age	< 0.0001	830.9	0.659	0.603–0.715	0.007
	T-tau	< 0.0001	632.5	0.884	0.856–0.912	< 0.0001
	P-tau181	< 0.0001	601.8	0.894	0.865–0.923	< 0.0001
	N-224+T-tau	< 0.0001	666.9	0.886	0.859–0.913	< 0.0001
	N-224+P-tau181	< 0.0001	651.7	0.895	0.866–0.924	< 0.0001
	N-224+*APOE*	< 0.0001	807.3	0.784	0.749–0.820	ns
	Aβ42+P-tau181	< 0.0001	593.3	0.921	0.902–0.940	< 0.0001

**Table 4:** Cohort 2. Alzheimer’s disease (AD) group compared to non-AD group. *p* values and Akaike information criterion (AIC) from binary logistic regression models. Area under the curve (AUC), 95% confidence intervals (95% CI) and differences between AUCs measured with DeLong test from receiver operating characteristic (ROC) analysis. *APOE* stands for presence of at least one *APOE* ε4 allele

	Cohort 2	*p*	AIC	AUC	95%CI	*p* value for difference with model containing N-224 alone
**AD vs. non-AD**	N-224	< 0.0001	423.5	0.808	0.760–0.855	–
	Age	< 0.0001	451	0.702	0.646–0.758	0.002
	T-tau	< 0.0001	284	0.905	0.875–0.936	< 0.0001
	P-tau181	< 0.0001	228.6	0.938	0.910–0.966	< 0.0001
	N-224+T-tau	< 0.0001	316.2	0.905	0.875–0.936	< 0.0001
	N-224+P-tau181	< 0.0001	266.5	0.936	0.907–0.965	< 0.0001
	N-224+*APOE*	< 0.0001	419.6	0.829	0.787–0.870	ns
	Aβ42+P-tau181	< 0.0001	257.9	0.932	0.905–0.958	< 0.0001

### Association of N-224 concentrations with MMSE

**Fig. 6:**
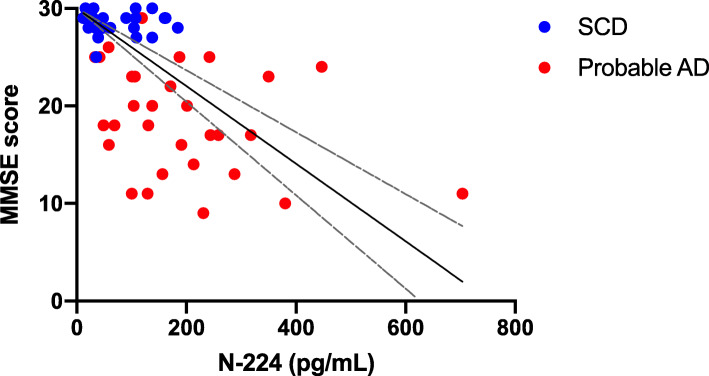
Correlation between N-224 concentrations (*x* axis) and Mini-Mental State Examination (MMSE) score (*y* axis). Linear regression line with 95% confidence intervals shown on top, as guidance

N-224 concentrations were inversely correlated to MMSE scores in cohort 1 (*r* = − 0.601, *p* < 0.0001) (Fig. 6, Table 5). N-224 concentration were associated to a larger deviation from MMSE = 30 (max score) at linear regression (*R*^2^ = 0.318, *β* = 0.564, *p* < 0.0001). Higher N-224 concentration could accurately indicate a pathological (< 24) MMSE score at binary logistic regression (*p* < 0.0001, odds ratio = 1.012) and ROC analysis (AUC = 0.827, 95% CI = 0.725–0.930) (Table 5).

**Table 5:** Association between N-224 concentrations and deviation from max Mini Mental State Examination (MMSE) score (= 30) at linear regression in subjective cognitive decline (SCD) and Alzheimer’s disease (AD) groups combined. Pathological MMSE score (< 24) was used as outcome in binary logistic regression and receiver operating characteristic (ROC) analyses. Correlation to MMSE is indicated by Spearman’s *r*

**Linear regression**	***R***^**2**^	***β***	***p***
Deviation from MMSE = 30	0.318	0.564	< 0.0001
**Binary logistic regression**	***p***	**Odds ratio**	
MMSE score < 24	< 0.0001	1.012	
**ROC analysis**	**AUC**	**95% CI**	
MMSE score < 24	0.827	0.725–0.930	
**Correlation**	***r***	***p***	
MMSE score	− 0.601	< 0.0001	

## Discussion

With this multicentre study, we validated the utility of the novel N-224 tau assay, previously shown as AD specific [3], in two independent clinical cohorts, and evaluated its potential to distinguish clinically diagnosed AD patients from controls, SCD, MCI and non-AD patients. In the present study, the N-224 tau fragment levels show good separation between AD and SCD/MCI and AD and non-AD, confirming that the increase in N-224 is especially linked to AD pathology as observed previously [3]. N-224 also showed a specific increase in AD compared to non-AD subgroups with parkinsonian disorders (PD, PDD, PSP, MSA).

### Strengths and limitations

This study was performed in two independent and well-characterized cohorts, including SCD patients and several types of non-AD dementias. The fact that we can confirm previous results showing low or normal concentrations of N-224 in several different non-AD groups, including a group as clinically relevant as SCD, adds to the confidence in the results of the current study.

We observed a correlation to age for tau core biomarkers and N-224, but this was only seen in diagnostic groups with no AD dementia, suggesting that when there is underlying AD pathology the increase in N-224 is specific and independent from age. All group comparisons were corrected for age and still showed significant differences between the groups. Age was also the weakest indicators of AD status in ROC and logistic regression analyses in cohort 2 (Fig. 5, Tables 3 and 4), although it is the strongest risk factor for AD. In cohort 2, the mean age of the participants was homogeneous, while in cohort 1, where age performed better than N-224 in indicating AD status, the mean age in the SCD group was lower than the probable AD group (57 vs. 69), which could represent a selection bias. Cohort 1 was also relatively small in size (30 SCD and 30 AD).

Higher N-224 concentrations were also associated with worse performance at MMSE, suggesting that baseline measurements could give insight on future cognitive outcome and encouraging the investigation of N-224 in longitudinal studies.

One of the main limitations of this study is that N-224 is not superior to core AD biomarkers in diagnostic accuracy for indicating AD status and was also not different between controls and SCD, while core AD biomarkers were. It has been shown that Aβ42 and tau become increasingly abnormal in CSF from SCD to progressively more severe stages of mild cognitive impairment (MCI) up to AD, but also that Aβ42 and tau biomarkers cannot differentiate between SCD and controls (reviewed in 14). Regarding tau specifically, a study has shown that increased CSF phosphorylated tau in healthy elderly could predict development of SCD at 3 years, but another study showed that while Aβ42 might decrease in SCD, T-tau and P-tau181 do not change significantly [15, 17]. In another study, plasma tau in SCD did not differ from levels in healthy controls, and plasma levels did not correlate with CSF [16]. In the present study, we observed that core AD biomarkers were lower (Aβ42) or higher (T-tau, P-tau181) in SCD compared to controls, although maintaining a large overlap between the two groups, while N-224 could not distinguish between controls and SCD. It seems therefore that, compared to core AD biomarkers, N-224 could either reflect neurodegeneration at later stages in those that will evolve to AD or remain generally stable in SCD, although longitudinal studies are required to confirm these observations.

Regarding non-AD dementias, one strength of the study is that we included a wide range of diseases, spanning from VaD to FTD as well as typical and atypical parkinsonisms (PSP, CBS, DLB, PDD). In these diseases, classic tau biomarkers are often normal and are not always of use in differential diagnosis [9–11]. Aβ42 showed varying diagnostic accuracy in atypical parkinsonisms, being especially decreased in DLB patients but not always differing from control groups (reviewed in [31]). In our study, P-tau181 was the most specific tau biomarker for AD. We have previously shown that N-224 was similarly low in tauopathies with usually normal CSF tau levels (PSP, CBD), but at the same time it did not correlate with the levels of T-tau, suggesting a different production pathway from T-tau in these diseases [3]. Here, N-224 correlated with T-tau and P-tau in all groups, but it was especially lower in typical and atypical parkinsonian disorders, as previously shown, suggesting that it might highlight a different pathological component in these diseases. This is also important in order to get information on the heterogeneity of tau in CSF. The N-224 assay could provide insight on tau metabolism and pathophysiology and potentially help discriminate between different tauopathies.

## Conclusions

Although not superior to classical tau biomarkers in diagnostic accuracy of AD, N-224 represents a useful assay to add to the tau biomarker toolbox to highlight the N-terminal component of tau in CSF. The present study encourages the wider use of a panel of assays directed to different tau fragments, as N-224 can distinguish subjects with AD from SCD and from subjects with typical and atypical parkinsonisms. N-224 could therefore potentially represent a further resource in the investigation of AD pathogenesis.

## Supplementary Information

**Additional file 1: Supplementary Table 1.** Correlations between N-224 and age, Aβ42 or Aβ42/40, T-tau and P-tau181 in cohort 1 (a) and cohort 2 (b). SCD = subjective cognitive decline, MCI = mild cognitive impairment, AD = Alzheimer’s disease.

## Data Availability

The datasets used and/or analysed during the current study are available from the corresponding author on reasonable request.

## Abbreviations

aa: Amino acid
Aβ: Amyloid β
AD: Alzheimer’s disease
AIC: Akaike information criterion
AUC: Area under curve
CBS: Corticobasal syndrome
CSF: Cerebrospinal fluid
CU: Cognitively unimpaired
DLB: Dementia with Lewy bodies
DSM-5: Diagnostic and Statistical Manual of Mental Disorders
ELISA: Enzyme-linked immunosorbent assay
FTD: Frontotemporal dementia
MCI: Mild cognitive impairment
MMSE: Mini Mental State Examination
MSA: Multiple system atrophy
NFT: Neurofibrillary tangles
PD: Parkinson’s disease
PDD: Parkinson’s disease dementia
PSP: Progressive supranuclear palsy
P-tau181: Phosphorylated tau
ROC: Receiver operating characteristic
SCD: Subjective cognitive decline
Simoa: Single molecule array
T-tau: Total tau
VaD: Vascular dementia
