# Supplementary material for: Cerebrospinal fluid N-224 tau helps discriminate Alzheimer’s disease from subjective cognitive decline and other dementias
Source: Alzheimers Res Ther. 2021 Feb 8;13:38. doi: 10.1186/s13195-020-00756-6 (PMC7871566; doi:10.1186/s13195-020-00756-6)

Supplementary table 1. Correlations between N-224 and age, Aβ42 or Aβ42/40, T-tau and P-tau181 in cohort 1 (a) and cohort 2 (b). SCD=subjective cognitive decline, MCI= mild cognitive impaiment, AD= Alzheimer’s disease


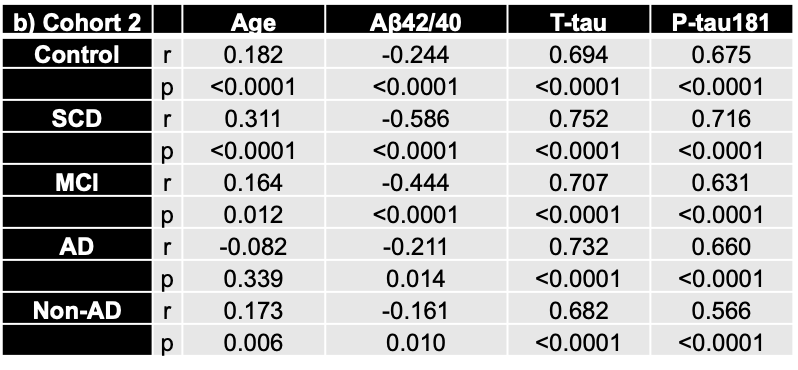

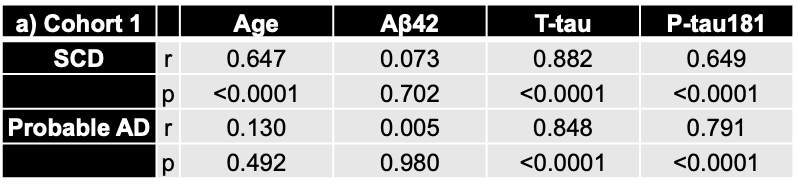

Supplement: Supplementary file 1 — Additional file 1: Supplementary Table 1. Correlations between N-224 and age, Aβ42 or Aβ42/40, T-tau and P-tau181 in cohort 1 (a) and cohort 2 (b). SCD = subjective cognitive decline, MCI = mild cognitive impairment, AD = Alzheimer’s disease. [file 13195_2020_756_MOESM1_ESM.docx]
